# 

**Published:** 1916-09-02

**Authors:** 


### Infantile Paralysis at Aberdeen.

Up to the present there is no reason to suppose that the terrible epidemic of poliomyelitis which is scourging New York has been imported into this country; and the Government authorities seem to be satisfied that there is little fear of such an event. At Aberdeen, as noted in The Hospital (July 29, p. 403), an outbreak has been going on for several weeks; but it is small indeed compared with the alarming outbreak in New York. The last report of the Medical Officer of Health for Aberdeen shows that twenty-six cases were notified during July. As mentioned in our previous note on this subject, ten of these were notified in the first week, so that only sixteen more took place in the remaining twenty-four days of the month, a distinct reduction in the average number of cases for the month of June and early days of July. It is gratifying also to learn that, though fatalities have occurred at Aberdeen, the general type of the disease is much less serious than in New York. In Manchester, we notice, four cases have been notified this year; but that is probably no more than the usual average—possibly even less—of a normal year.

### The Book Market.

LIST OF NEW BOOKS RECEIVED FROM THE FOLLOWING PUBLISHERS:—

Walter Hill: "The Holidays: Where to Stay and What to See." 1s. net.

John Bale, Sons and Danielsson, Ltd.: "Bed-Sores: Their Prevention and Cure." By Catherine W. Smart. 1s. net.

Cambridge University Press: "Biometrika." A Journal for the Statistical Study of Biological Problems. May 1916. 10s.

RATES OF SUBSCRIPTION: Twelve months, 6/6; Six months, 4/-; Three months, 2/-, post free to any address in the United Kingdom. Abroad, Twelve months, 8/8; Six months, 5/-; Three months, 2/6, post free. THE HOSPITAL "Index" to Volume LIX., October 1915 to March 1916, is now ready, price 1½d. per copy, post free. Address The Manager, "THE HOSPITAL," The Hospital Building, 28/29 Southampton Street, Strand, London, W.C.

